# Supplementary material for: Neuroimaging findings and balance problems after mild traumatic brain injury: A systematic review protocol
Source: PLoS One. 2025 Feb 5;20(2):e0307339. doi: 10.1371/journal.pone.0307339 (PMC11798431; doi:10.1371/journal.pone.0307339)
Supplement: S1 Appendix — (DOCX) [file pone.0307339.s002.docx]

### **Appendix 1 Search terms used in preliminary PubMed literature search (6/3/2024).**

| **Search name** | **Results** | **Query** |
| --- | --- | --- |
| All combined | 118 | (("brain concussion"[MeSH Terms] OR "mTBI"[Title/Abstract] OR "concussion*"[Title/Abstract] OR "mild traumatic brain injur*"[Title/Abstract] OR "Mild traumatic brain injury"[Title/Abstract:~2] OR "Mild traumatic brain injuries"[Title/Abstract:~2] OR "brain injuries, traumatic"[MeSH Terms]) AND ("Neuroimaging"[MeSH Terms] OR "Electroencephalography"[MeSH Terms] OR "Magnetoencephalography"[MeSH Terms] OR "Diffusion Tensor"[Title/Abstract] OR "DTI"[Title/Abstract] OR "Diffusion Tractography"[Title/Abstract] OR "Diffusion-Weighted Imaging"[Title/Abstract] OR "DWI"[Title/Abstract] OR "magnetic resonance imag*"[Title/Abstract] OR "MRI"[Title/Abstract] OR "Tomography"[Title/Abstract] OR "FMRI"[Title/Abstract] OR "electroencephalogra*"[Title/Abstract] OR "EEG"[Title/Abstract] OR "magnetoencephalogra*"[Title/Abstract] OR "MEG"[Title/Abstract] OR "white matter hyperintensit*"[Title/Abstract]) AND ("Vertigo"[MeSH Terms] OR "Postural Balance"[MeSH Terms] OR "Dizziness"[MeSH Terms] OR "Vertigo"[Title/Abstract] OR ("postur*"[Title/Abstract] AND ("equlibrium*"[Title/Abstract] OR "control*"[Title/Abstract] OR "balance*"[Title/Abstract] OR "stab*"[Title/Abstract])) OR "balance"[Title/Abstract] OR "stability"[Title/Abstract])) ) |
| Outcome | 577,538 | **("Vertigo"[MeSH Terms] OR "Postural Balance"[MeSH Terms] OR "Dizziness"[MeSH Terms] OR "Vertigo"[Title/Abstract] OR ("postur*"[Title/Abstract] AND ("equlibrium*"[Title/Abstract] OR "control*"[Title/Abstract] OR "balance*"[Title/Abstract] OR "stab*"[Title/Abstract])) OR "balance"[Title/Abstract] OR "stability"[Title/Abstract]))** |
| Exposure | 734,417 | **("Neuroimaging"[MeSH Terms] OR "Electroencephalography"[MeSH Terms] OR "Magnetoencephalography"[MeSH Terms] OR "Diffusion Tensor"[Title/Abstract] OR "DTI"[Title/Abstract] OR "Diffusion Tractography"[Title/Abstract] OR "Diffusion-Weighted Imaging"[Title/Abstract] OR "DWI"[Title/Abstract] OR "magnetic resonance imag*"[Title/Abstract] OR "MRI"[Title/Abstract] OR "Tomography"[Title/Abstract] OR "FMRI"[Title/Abstract] OR "electroencephalogra*"[Title/Abstract] OR "EEG"[Title/Abstract] OR "magnetoencephalogra*"[Title/Abstract] OR "MEG"[Title/Abstract] OR "white matter hyperintensit*"[Title/Abstract])** |
| Participants | 27,653 | **(("brain concussion"[MeSH Terms] OR "mTBI"[Title/Abstract] OR "concussion*"[Title/Abstract] OR "mild traumatic brain injur*"[Title/Abstract] OR "Mild traumatic brain injury"[Title/Abstract:~2] OR "Mild traumatic brain injuries"[Title/Abstract:~2] OR "brain injuries, traumatic"[MeSH Terms])** |

### 
