# Supplementary material for: Neuroimaging findings and balance problems after mild traumatic brain injury: A systematic review protocol
Source: PLoS One. 2025 Feb 5;20(2):e0307339. doi: 10.1371/journal.pone.0307339 (PMC11798431; doi:10.1371/journal.pone.0307339)
Supplement: S2 Appendix — (DOCX) [file pone.0307339.s003.docx]

### **Appendix 2 List of variables for data extraction.**

|  | **Topic** | **Specific** |  |
| --- | --- | --- | --- |
|  | Study | Author |  |
|  |  | Year of publication |  |
|  |  | Name of publication |  |
|  |  | Study design |  |
|  | Participants | Mean, range of patient ages |  |
|  |  | Time between injury and scan |  |
|  |  | Sex |  |
|  |  | Most prevalent cause of injury |  |
|  |  | Loss of consciousness | |
|  | Symptom | How balance problem diagnosed |  |
|  |  | Time balance problems in study population studied |  |
|  | Treatment groups (if relevant) |  |  |
| **OUTCOME SPECIFIC- Balance symptom presentation, persistence** | Sample size, effect size, error term; number missing | |  |
|  | Brain region | As indicated in study |  |
| Per brain region | Neuroimaging modality | MRI (volumetrics, lesions), fMRI, DWI/DTI, EEG, MEG |  |
| Per neuroimaging modality | Metrics | Conventional MRI - brain volume (mm^3^), white matter lesion load or volume |  |
|  |  | DWI/DTI - mean diffusivity, fractional anisotropy, axial diffusivity, radial diffusivity, diffusion kurtosis |  |
|  |  | fMRI - functional activation during task, resting state functional connectivity, T scores |  |
|  |  | EEG- event-related potential component amplitude |  |
|  |  | MEG- slow wave |  |
