# Supplementary material for: Neuroimaging findings and balance problems after mild traumatic brain injury: A systematic review protocol
Source: PLoS One. 2025 Feb 5;20(2):e0307339. doi: 10.1371/journal.pone.0307339 (PMC11798431; doi:10.1371/journal.pone.0307339)
Supplement: Attachment — Submitted filename: Response to Reviewers.docx [file pone.0307339.s004.docx]

Dear Editors.

Below is a list of the critiques and questions brought up in your review. In red is a copied and pasted version of the various concerns and questions. In black text below each question is our response.

Thank you very much for your thorough work and contribution to the peer review process. I look forward to further comments.

A rebuttal letter that responds to each point raised by the academic editor and reviewer(s). You should upload this letter as a separate file labeled 'Response to Reviewers'

1. When submitting your revision, we need you to address these additional requirements.

   Please ensure that your manuscript meets PLOS ONE's style requirements, including those for file naming. The PLOS ONE style templates can be found at
   https://journals.plos.org/plosone/s/file?id=wjVg/PLOSOne_formatting_sample_main_body.pdf and
   <https://journals.plos.org/plosone/s/file?id=ba62/PLOSOne_formatting_sample_title_authors_affiliations.pdf>
   a. I have completed the above, adjusting the manuscript title page, body, and references according to the links provided above.
2. Please include captions for your Supporting Information files at the end of your manuscript, and update any in-text citations to match accordingly. Please see our Supporting Information guidelines for more information: <http://journals.plos.org/plosone/s/supporting-information>.
   a. Appendices 1 and 2 removed from main manuscript and added as separate files with titles according to above link.
3. Please review your reference list to ensure that it is complete and correct. If you have cited papers that have been retracted, please include the rationale for doing so in the manuscript text, or remove these references and replace them with relevant current references. Any changes to the reference list should be mentioned in the rebuttal letter that accompanies your revised manuscript. If you need to cite a retracted article, indicate the article’s retracted status in the References list and also include a citation and full reference for the retraction notice.
   a. Reference list adjusted to fit recommended formatting. Parentheses replaced with brackets, and all are in ascending order of appearance.
4. Reviewer #4: Dear Authors, thank you for your contribution.

   I would like to know the purpose of publishing a Systematic Review and Meta-Analysis protocol without having done it.
   a. This is an excellent question. The purpose of publishing a systematic review or meta analysis protocol is to ensure transparency, minimize bias, and perhaps most importantly add to the literature regarding how to perform a meta analysis with difficult to compare modalities. For example, this study is comparing imaging modalities, and our protocol outlines how to compare the variable of the various modalities. This may prove useful for others who desired to perform studies looking at different pathology comparing findings from different imaging modalities.
5. Reviewer #5: The protocol is sound. The only obvious issue is that there is no statement identifying where the data for the study will be made available. This wouldn't be an issue until publication of the article if the protocol were not being independently published first. In that case, question 4 above (Have the authors described where all data underlying the findings will be made available when the study is complete?) must be answered no. Adding this statement should be very easy. I do have clinical questions about their methodology, such as the decision to limit analysis to mild TBI, but that should not hinder the progress of their protocol. Beyond that, the study protocol largely meets the expectations of the journal and should be allowed to proceed.
   a. Yes, the data is currently being collected, and thus this question likely does not apply to the publication of a protocol. Please advise if it would be prudent to add an addendum when the data is available. We would be more than happy to comply.
   b. Regarding your question on why we are just limiting our study to mTBI, that is because mTBI and TBI are differentiated historically by imaging findings. TBI has significant imaging findings, while mTBI is generally characterized by no imaging findings. Of course, this study is examining the truth to this assumption, finding that long term complications after mTBI do indeed have subtle imaging findings in the long term. We also chose just mTBI in order to keep the scope narrow enough to be manageable for data acquisition and management purposes.
